# Macronutrients, Micronutrients, and Malnutrition: Effects of Nutrition on Immune Function in Infants and Young Children

**DOI:** 10.3390/nu17091469

**Published:** 2025-04-26

**Authors:** R. J. Joost van Neerven

**Affiliations:** Cell Biology and Immunology, Wageningen University & Research, 6708 WD Wageningen, The Netherlands; joost.vanneerven@wur.nl

**Keywords:** macronutrients, micronutrients, vitamins, minerals, infants, children, oligosaccharides, milk, formula feeding, malnutrition, immune status, infection, allergy

## Abstract

The function of the immune system is not only dependent on factors like genetics, age, the environment, and exposure to infectious agents and allergens but also on our microbiota and our diet. It has been known for centuries that food can influence health and vulnerability to infection. This is especially true for infants, young children, and the elderly. This review focuses on how nutrition can support immune function from gestation to school-aged children. Immune support begins during pregnancy by the mother’s diet and transfer of nutritional components as well as antibodies to her fetus. After birth, breastfeeding is of crucial importance for immune development as well as for the development of the intestinal microbiota of an infant. If breastfeeding is not possible, early-life formulas are an alternative. These can provide several of the functionalities of breastmilk, as well as the key nutrients a child needs. New foods are introduced during and after weaning, and after this period, children switch to consuming a normal diet. However, due to circumstances, children can be malnourished. This can range from severe protein/energy malnutrition to micronutrient deficiencies and obesity, all of which can affect the function of the immune system. This narrative review describes the immune challenges in early life, explores breastfeeding and early life nutrition, and provides mechanistic insight into the relative contribution of macronutrients, micronutrients and other immunomodulatory food components that can support immune function in early life.

## 1. Introduction

Since ancient times, the link between food and health has been noted. For example, anti-inflammatory and fever-reducing components from willow bark and ginseng and anti-infective and wound-healing effects of milk and colostrum have been used through the ages. While the old Hippocratian adage of the interconnectedness of food and medicine was historically pervasive, this has been penned more than 200 years after his death. Despite its perceived authenticity, it does, however, indicate that people realized long ago that food components can profoundly contribute to immune function and organismal health. In addition, the transfer of immunity and protection against infections by breastfeeding is well established, and many food components that support immune function are currently available.

It is now well established that food plays an important role in the support and normal functioning of our immune system. Firstly, breastfeeding—or, in its absence, early-life nutrition—supports the development of the immune system and provides protection against infections though its immunomodulatory components. Further, micronutrients (vitamins and minerals) are essential for the normal functioning of the immune system. Finally, new immunomodulatory components as well as microbiota-targeting components have been identified and are also applied in foods and dietary supplements to support immune function.

Despite progress in medicine and nutrition, infants and children in developing countries still face high child infection and mortality rates, and in Western countries, allergies and metabolic diseases affect child health. These differences are linked to an unbalanced distribution of wealth, healthcare, education, and food in the world. In addition, urbanization, changing dietary habits, increased travel, and changes in the environment all affect the development of allergies and the spread of infectious diseases.

Rather than treating these diseases, preventive strategies may help to reduce the impact of these diseases. Nutrition, dietary status, and malnutrition play important roles in many diseases and conditions, either directly or indirectly via the intestinal microbiota. Therefore, our diet may offer opportunities to reduce the prevalence of childhood diseases, especially in relation to the immune-supporting effects of nutrition. The promotion of breastfeeding, improving early-life nutrition formulas, preventing micronutrient deficiencies and malnutrition, and early introduction of potentially allergenic foods in diet, can contribute to lowering the prevalence of infections and allergies in children. In addition, dietary changes can help reduce clinical symptoms in food allergies, intolerance, and IBD. Gluten-free diets can even resolve inflammation, resulting in complete remission in children with celiac disease.

The immune system protects the body against external (infections and allergies) and internal threats (cancer). A well-balanced immune response to these challenges is of key importance for good immune health. In contrast, excessive, prolonged immune responses can lead to allergies and (chronic) inflammatory conditions that are relevant for most age-related non-communicable diseases, and finally, inadequate immune responses increase susceptibility to infections and cancer.

The action of the immune system is not only determined by many factors, such as one’s genetic makeup and exposure to pathogens, allergens, tumor cells, and tissue damage but also to environmental exposures, diet, and the intestinal microbiota. The functioning of the immune system has been shown to change over our life time, with young children having a relatively immature immune system in the first 3 years of life [1,2]. Similarly, the immune system in ageing people is subject to immune senescence, which is associated with a reduced flexibility of adaptive and innate immune responses to infection as well as inflammaging that is associated with chronic diseases [3,4,5,6,7,8].

Therefore, children of 0–5 years old and elderly people >65 years old are more prone to gastrointestinal and respiratory infections compared to healthy adults [9,10]. In addition, in the first year of life, a bias towards pro-allergic Th2 responses is present, with antimicrobial Th1 and pro-inflammatory responses being underdeveloped [1,11], potentially explaining why allergic sensitization often occurs in infancy and childhood.

These findings indicate that the very young (0–5 yr) and the old (>65 yr) in particular may benefit from immune support via nutrition. Dietary components can modulate the function of the immune system either directly or via the modulation of the intestinal microbiota and the metabolites they produce. The molecular mechanisms by which bacterial metabolites and specific food components interact with receptors in the immune system were reviewed recently [12].

A scientific basis for the link between nutrition and health has been provided over the last 150 years by key observations on the transfer of immunity by breastfeeding by Paul Ehrlich [13], the modulation of the intestinal microbiota by fiber, prebiotics and the effects of probiotics [14,15], the importance of micronutrients in the function of the immune system [16], and the effects of the Western diet on fueling inflammatory disorders [17,18,19]. These findings have led to recommendations to increase breastfeeding; the advice for high-fiber intake through vegetables, fruits, and other foods; an increased consumption of fatty fish (such as salmon and some tuna and mackerel) with *n*-3 fatty acids; and a balanced intake of macro- and micronutrients. This narrative review provides an overview of our knowledge on the effects of macronutrients, micronutrients, as well as malnutrition on the immune function in infants and young children, affecting their susceptibility to infections and allergies.

## 2. Infections, Allergies, Gastrointestinal Inflammation, and Malnutrition in Children

### 2.1. Infections

It is well established that children under 5 and ageing people over 65 years of age are more susceptible to infections compared to healthy adults [1,11]. For example, influenza and bacterial respiratory infections lead to increased mortality in these age groups [9,10]. Similarly, the age effect can also be seen for diarrhea-related mortality, although the age dependency is a bit less conclusive [20]. It is thus not unexpected that in children under the age of 5, infectious diarrhea and pneumonia are major causes of child death [21].

In addition, there is a striking difference between diarrheal deaths in low-income vs. high-income countries. In Africa and Southeast Asia in particular, the percentage of children under the age of 5 that die because of diarrhea is very high (approximately 20% or more of all child deaths under the age of 5).

### 2.2. Food and Respiratory Allergies

In Westernized countries, childhood allergies also have a high prevalence. This illustrates that it is very important that the immune system in early life is capable of dealing with infectious challenges, and that it also does not develop Th2 responses that drive allergic immune responses.

The development of allergies typically occurs early in life [22] with eczema and food allergies preceding the development of asthma and hay fever [23]. Over the last 30–40 years, the prevalence of allergies has increased dramatically. This was first noted for asthma [24] but is also seen for food allergies and hay fever [25]. This trend that occurs within a lifespan is indicative of a crucial role for environmental factors, as it is too short for a genetic cause. The environmental factors that have been identified as possible explanations for this increase in allergies are urbanization, farming, pollution, family size, and antibiotic use, as well as to changes in diet and microbiota [26,27,28].

A Westernized diet; the consumption of highly processed foods; a decreased consumption of fiber, fresh vegetables, and fruit; and changes in microbiota composition have all been linked to the increased prevalence. Interestingly, even though this trend was first noted in Western high-income countries, these increases are now also evident in other regions in the world, again suggesting a link to changing environments and dietary habits.

### 2.3. Celiac Disease and IBD in Children

In contrast to allergies, many chronic inflammatory diseases are linked to ageing, with the exception of celiac disease, a food-related intestinal inflammatory disease, and inflammatory bowel disease (IBD, consisting of Crohn’s disease and Ulcerative Colitis).

These two types of chronic gastrointestinal inflammation that occur relatively frequently in children. Celiac disease (CD) is a chronic gluten-induced gastrointestinal disease that can have gastrointestinal as well as systemic symptoms. The prevalence of CD is around 1%, with a higher prevalence in children compared to adults [29]. CD is diagnosed by the detection of the destruction of small intestinal villi in biopsies and by the detection of transglutaminase 2-specific IgA antibodies [30]. In CD, deamidated gluten peptides are recognized by CD4+ T cells in the context of HLA-DQ2 and DQ8 [31]. Symptoms can be quite diverse and not limited to the gastrointestinal tract, and management of the disease is mainly based on a complete gluten-avoidance diet. The symptoms of CD are fully reversible after adapting to a gluten-free diet.

In contrast, the prevalence of IBD worldwide is <0.1% and is mainly seen in children in Europe, the USA, and Canada [32]. Although diets for the management of IBD are under investigation [33], IBD is primarily managed clinically, so we will not go in more detail here.

### 2.4. Malnutrition

Another important condition affecting the function of the immune system in children is malnutrition. Malnutrition is an overarching definition of a wide range of conditions, presenting as wasting, stunting, being underweight, obesity, double burden (stunting combined with overweight), and severe protein–energy malnutrition (Kwashiorkor or Marasmus) [34].

The occurrence of malnutrition is linked to many different causes, including one’s socioeconomic status, age, education level, gender, family size, and geographical location [35]. The prevailing consensus, however, is that poverty is the main cause of malnutrition [36]. Poverty is not only linked to the availability of food but also other factors that affect children’s health, like access to clean drinking water, access to healthcare, and the choice to stop breastfeeding in order to be able to work and earn an income. Overall, malnutrition can have many effects on the normal functioning of the immune system [36]. In addition, carbohydrate deficiencies and essential fatty acid deficiencies can occur.

Micronutrient deficiencies occur relatively often. These micronutrient deficiencies do not directly lead to a visible phenotype but increase susceptibility to infections. Even though children may not present as being malnourished, a lot of children across the globe have micronutrient deficiencies [37]. A micronutrient deficiency can be limited to only vitamin D deficiency or vitamin B12 deficiency in some countries, but in some countries, up to 40% of young children have >2 deficiencies simultaneously. The prevalence of these deficiencies in low-income countries can be as high as 70–80% of all children between 6 months and 5 years of age [37].

A nutritional survey in Southeast Asia, the SEANUTS study and its follow-up, provided an overview of the micronutrient status in about 13,000 children from 6 months to 12 years of life [38,39,40,41,42,43,44]. These studies showed that vitamin A and vitamin D, which are typical vitamins that have the strongest link to immune function, deficiencies occur both in urban and in rural areas, and around 30% of children have vitamin D deficiency. Interestingly, these deficiencies are more common in urban areas than in rural areas, suggesting differences in diet.

Overall, up to 50% of young children in many parts of the world are suffering from at least one micronutrient deficiency, even in high-income countries, although the prevalence of micronutrient deficiency is lower there. Even though not all effects of malnutrition (like stunting) can be corrected, all of the above can be prevented by providing access to a well-balanced diet to young children [45].

Finally, another form of malnutrition that affects immune function is obesity. The prevalence of overweight and obese children is also increasing worldwide, and this is typically driven by an excessive intake of food [46,47,48,49].

Although differences exist on exactly how the immune system is affected, all types of malnutrition increase susceptibility to infections, as reviewed elsewhere [36,48].

## 3. Immunomodulation by Food

**Figure 1:**
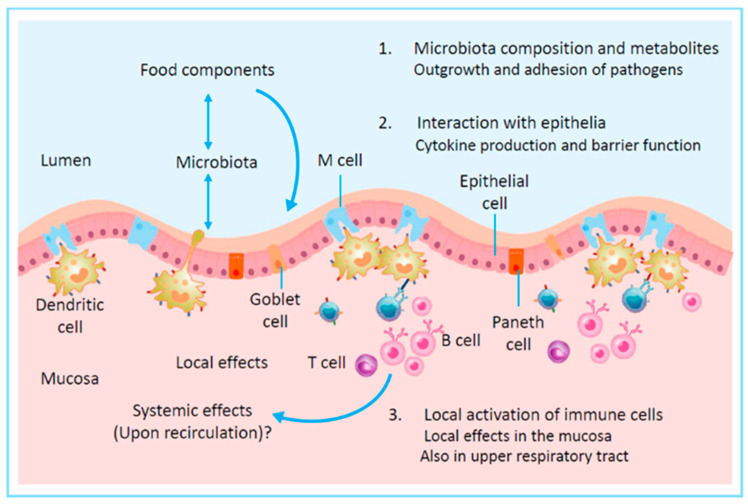
The ways in which food components can have an effect on the immune system. Figure adapted from [50].

Food can affect the immune system via several different routes [50]. As shown in Figure 1, food can influence the intestinal microbiota composition as well as the metabolites they produce. A second effect food components can have acts via the intestinal epithelium, which can produce cytokines and chemokines and attract immune cells to the tissue. The barrier function of intestinal epithelium is also very important to prevent leakage and transcytosis of bacteria(l components), viruses, and food allergens into the underlying tissue. Further, food can directly interact with the cells of the immune system in the intestinal tissues via sampling of the intestinal content by dendritic cells and M cells that can then initiate immune responses. These effects are mainly local, although systemic effects have also been observed. Finally, food components that become systemically available via uptake in the bloodstream can support immune function at distant sites throughout the body (typically, small components like micronutrients). In addition, food components can potentially also come into direct contact with the immune system that links to the respiratory tract via the tonsils [51].

**Figure 2:**
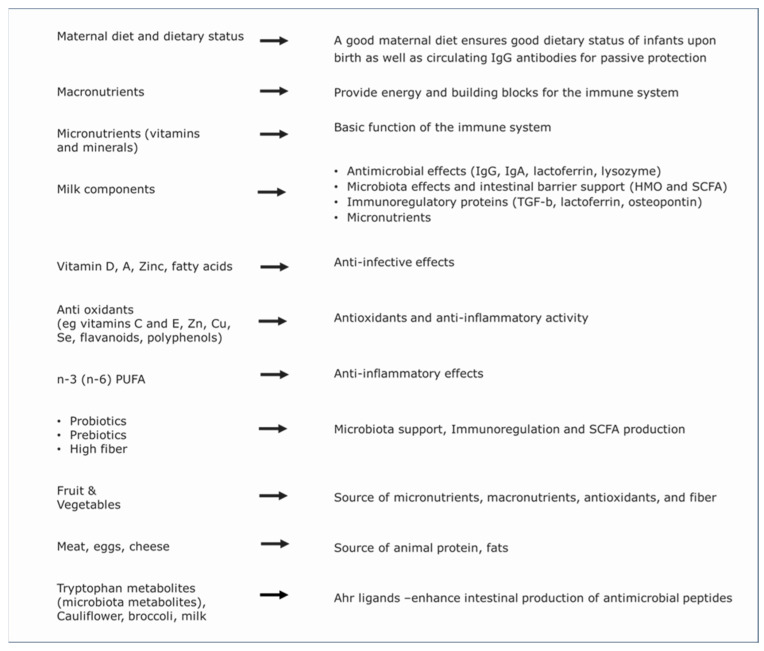
Overview of different food components and their main effects on the immune system.

The next section of this review will focus on the different foods (components) and the effects they can have, as summarized in Figure 2.

### 3.1. Nutritional Immune Support in Infants and Children

The impact of macronutrients (proteins, carbohydrates, and fats); micronutrients (vitamins and minerals); and also other specific food components, like anti-oxidants, flavonoids, non-digestible carbohydrates, PUFA, as well as microbiota-related prebiotics, probiotics, and short-chain fatty acids (postbiotics) on the immune system has been reviewed elsewhere recently and will not be discussed here in detail [12,16,51,52,53,54,55].

#### 3.1.1. Macronutrients

Macronutrients typically provide the body with building blocks for the formation of cellular components, energy, and protein synthesis that are important for growth, normal function of the body, as well as in immune responses. In addition, some macronutrients are used for the generation of chemicals in the immune response (e.g., nitric oxide and lipid inflammatory mediators).

A large part of the function of the immune system is mediated by proteins. Protein intake is crucial, because proteins contain amino acids that the body can use after digestion to generate new proteins. Some amino acids can be produced by the human body, but other amino acids cannot. These are the essential or conditionally essential amino acids. Conditionally essential amino acids are essential only in illness and stress or when more of these amino acids are needed for other reasons. For example, in phenylkentonuria, the enzyme that converts phenylalanine into tyrosine (phenylalanine hydroxylase) is deficient, making tyrosine in the diet essential. As proteins are made of combinations of all amino acids, protein quality in a diet is dependent on the intake of sufficient intake levels of especially the (conditionally) essential amino acids. These are higher in animal proteins compared to plant proteins [56].

#### 3.1.2. Micronutrients

Micronutrients can regulate many aspects of the basic function of the immune system’s response (e.g., iron, zinc, vitamin A, and vitamin D). In addition, some components like vitamin D and zinc can have a direct anti-infection effect. It is known from the literature that a deficiency of micronutrients is linked to increased susceptibility to infections [16,57,58,59]. A recent meta-analysis on the effects of nutritional supplements showed protective effects of zinc and vitamin D on viral respiratory infections in different ages and continents [60].

As a result of this, micronutrients are currently the only food components of which health claims in relation to the normal function of the immune system can be used according to the European Food and Safety Agency (EFSA) (see links in Table A1). Regulation for health claims on foods is very strict (in Europe), and there has to be broad scientific consensus as well as causality in order to be able to make these claims. None of the other food components discussed here, although quite some scientific data is present on their effects, have a level of substantiation that is high enough to carry such health claims. They may, however, in part, already be included in dietary guidelines.

### 3.2. Maternal Nutrition, Breastfeeding, Cow’s Milk, and Infant Nutrition

#### 3.2.1. Maternal Nutrition

Nutrition affects not only the function but also the development of the immune system and the risk of developing infectious diseases, especially in the first days and months of life. The role of maternal nutrition in this is becoming clearer, as one’s maternal nutritional status will not only influence the composition and quality of breastmilk [61] but also the nutritional status of the infant just after birth [62], as well as the infant’s microbiota composition [63,64]. Breastmilk composition has clear effects on the infant’s immune system, as reviewed elsewhere by [65,66].

Most of the studies on maternal diet and breastfeeding on the immune development of infants have focused on the effects on allergic diseases, and the positive impact of maternal intake of polyunsaturated fatty acids and vitamin D on allergy has been demonstrated [64,67,68,69,70,71,72,73]. Effects of probiotic and prebiotic supplementation of the maternal diet are not well established yet [72,74]. Based on current knowledge, a maternal dietary approach that may reduce the risk of developing allergies has been reported [75].

In addition to micro- and macronutrient transfer from mother to fetus during pregnancy, immunoglobulin G (IgG) is also transferred via the placenta during pregnancy [76,77]. These IgG antibodies offer passive protection to the infant upon birth before it can efficiently produce these antibodies itself. Breastfeeding provides IgA antibodies in the gastrointestinal tract to specifically provide gastrointestinal protection against infections.

However, the IgG antibodies from the mother that are transferred into the circulation of the infant decrease gradually during the first months after birth.

#### 3.2.2. Breastfeeding

Exclusive breastfeeding is recommended for the first six months of life by the WHO and UNICEF [78]. Breastfeeding has been termed nature’s first functional food in science [79]. Breastfeeding has two important functions: it not only provides nutrients to support growth and development, but it also provides protection when the infant’s immune system is not fully developed. Indeed, immunity can be transferred by milk from mother to child by breastfeeding [13].

It is well established that breastfeeding offers protection against infections [80,81,82,83]. This extends beyond gastrointestinal infections, also protecting against respiratory infections and mortality [84,85,86]. The role of breastfeeding in relation to allergy is less clear and may be more dependent on the exact composition of the breastmilk [65,87,88].

Even though the innate immune system is relatively mature upon birth, the adaptive immune system still needs to develop during the first 2–3 years of life in order to produce sufficiently high T-cell and antibody responses upon infection. Infants are therefore quite dependent on passive immune support through the antibodies they have received via the placenta during pregnancy, as well as the IgA antibodies (and some IgG) that are provided during breastfeeding.

Human breastmilk contains macronutrients, like (bioactive) proteins, fats and lipids, sugars (human milk oligosaccharides and lactose), and micronutrients (vitamins and minerals). Additional components are maternal immune cells, hormones, water, and some bacteria—although there is still discussion on whether these are present in the milk or are a result of contamination after extrusion of the milk (see Table 1).

Many of these components play a role in supporting immune functions, including antibodies (mainly IgA and some IgG); antimicrobial enzymes and -proteins (lactoferrin, lactoperoxidase, and lysozyme); human milk oligosaccharides (HMOs; >200 different HMOs); anti-inflammatory proteins (TGF-β and IL-10); immunomodulatory components (lactoferrin, osteopontin, and extracellular vesicles); and components with antiviral activity like the milk fat globule-associated MFG8 and lactoferrin [65,82,89,90].

One of the key factors in breastmilk that promote passive protection to infants turned out to be IgA antibodies [2,76,77]. IgA antibodies are mostly found on mucosal surfaces, like the intestinal tract and the respiratory tract, and are ingested during breastfeeding. IgA antibodies reduce intestinal colonization by pathogens, limit microbial translocation, and enhance immune sampling of the intestinal microbiota [76].

Together, the components in breastmilk can support basic function of the immune system (micro- and macronutrients), offer passive protection (IgA and IgG), manage microbial growth with anti-bacterial and antiviral components, support a healthy microbiota by the HMO that promote growth of Bifidobacteria and Lactobacilli (that have the enzymes to digest HMO), support the production of SCFAs, support intestinal barrier function, and finally, prevent excessive immune activation by anti-inflammatory components (TGF-β and low levels of IL-10). The latter are also important for the development of oral tolerance to food proteins, as well as being non-responsive to microbiota. Without this tolerance, intestinal inflammation would occur in response to dietary proteins and the commensal microbiota. Therefore, breastmilk is a well-balanced package of growth and immune-supporting nutrients.

#### 3.2.3. Cow’s Milk and Infant Formula

The composition of cow’s milk is different from human milk, but most of the components present in breastmilk are also present in cow’s milk, albeit at different levels [90] (Table 1). A series of epidemiological studies has demonstrated a protective effect of the farming environment in relation to allergies [91,92,93]. This effect is not only linked to the consumption of raw milk, but also to the exposure to bacterial components in farm dust. Several studies over the past two decades have shown an inverse correlation between the consumption of unpasteurized (raw) cow’s milk and the occurrence of respiratory allergies [91,93,94,95,96,97,98,99] and possibly also respiratory infections [100].

Heat treatment of milk, however, ablated this effect [97], indicating that proteins as heat sensitive components of milk may play an important role in this [90]. The identification of the milk proteins that could potentially reduce the risk of developing allergies (and need to be preserved if provided) is important, as this may help to provide this effect of raw milk in a safe way in early-life nutrition.

Indeed, several bovine milk proteins have been shown to have effects on cells of the human immune system in in vitro studies, including the anti-inflammatory cytokines IL-10 [101] and TGF-β [102], lactoferrin [103], and bovine IgG [104]. In addition, bovine milk contains other components that can modulate immune function, like the milk fat globular membrane, 3-and 6- sialyllactose, extracellular vesicles, alkaline phosphatase, and osteopontin.

**Table 1:** Immunologically active components in breastmilk, cow’s milk, and infant and toddler nutrition (IFT).

Component	Function	Ratio Breastmilk/Cow’s Milk *	Present in IFT
Immunoglobulins (IgA, IgG, IgM) ^#^	Anti-infective	IgA: 10 IgG: 0.1 IgM: 1.1	Inactivated Supplemented in some IFTs ^+^
Lactoferrin	Anti-microbial Anti-inflammatory	20	Inactivated Supplemented in several IFT
Lactoperoxidase	Anti-microbial	0.1	Inactivated
TGF-β1 ^+^, TGF-β2 ^+^	Anti-inflammatory Strengthening intestinal barrier	TGF-β1: 0.2 TGF-β2: 0.2	Yes, TGF-b is quite heat resistant
IL-10 ^+^	Anti-inflammatory	6	Inactivated
Pro-inflammatory cytokines (IL-6, IL-1β, TNF-α) ^+^	Anti-infective Pro-inflammatory	IL-1β: 0.4 IL-6: 1 TNF-α: 0.2	Inactivated
MFGM: MFGE8/Lactadherin	Anti-viral	3	Supplemented in several IFT
MFGM: Xantine oxidase	Anti-microbial	1	Supplemented in several IFT
Osteopontin	Anti-inflammatory	7	Supplemented in some IFTs
Vitamin A, C, D and other micro-nutrients	Basic immune function (all) anti-oxidant (vitamins) anti-infective (some e.g. vit D)	Vitamin A: 2.2 Vitamin C: 1 Vitamin D: 3	Vit A and D supplemented to all IFT (based on regulations)
HMO	Shaping of microbiota, induction of SCFA Anti-inflammatory (SCFA and directly) Strengthening intestinal barrier (SCFA)	3′SL: 3.8 6′SL : 21 All HMO: 70	2′FL, 6′SL, LNnT supplemented to some IFT
Prebiotics	Shaping of micro-biota & induction of SCFA Strengthening intestinal barrier (SCFA) Anti-inflammatory (SCFA)	n/a	Supplemented to most IFT
*n*-3 PUFA (DHA, ARA, EPA)	Anti-inflammatory	DHA: 25 ARA: 2.3 EPA: 2.4	Supplemented in IFT
Probiotics	Gut health, immune support	n/a	Probiotics added to some formula
Extracellular vesicles	Anti-inflammatory Strengthening intestinal barrier	Similar ^X^	Inactivated May be present in some ingredients though
Alkaline phosphatase ^+^	Inactivation of LPS Antiinflammatory	Similar ^X^	Inactivated

The information in this table is based on [50,51,90,101,103,105,106,107,108,109,110,111,112,113]. During milk processing, many proteins are functionally inactivated (denatured), and they are present as denatured proteins in IFT, still having their nutritional value. * Concentration ratios based on [90]; ^#^ Human: IgA >> IgG > IgM; Cow: IgG > IgA > IgM; ^+^ Very low concentrations; ^X^ No numbers available.

Whereas some factors in human and bovine milk are present at comparable levels, some components are much higher in breastmilk (see Table 1). These components are the human milk oligosaccharides; lactoferrin; and *n*-3 polyunsaturated fatty acids (*n*-3 PUFA), like EPA and DHA. These are components that are now often added to infant nutrition to make up for this difference (Table 1).

An important notion in relation to infant nutrition is that almost all milk proteins, with TGF-β as an exception, are fully denatured during the production process, thus inactivating them. For this reason, minimal processing technologies are investigated to prevent this in future infant formula products. In addition, minimal processing can also prevent glycation of proteins, thus reducing their immunogenicity and allergenicity.

### 3.3. Effects of Microbiota and Their Metabolites on Immune Function

**Figure 3:**
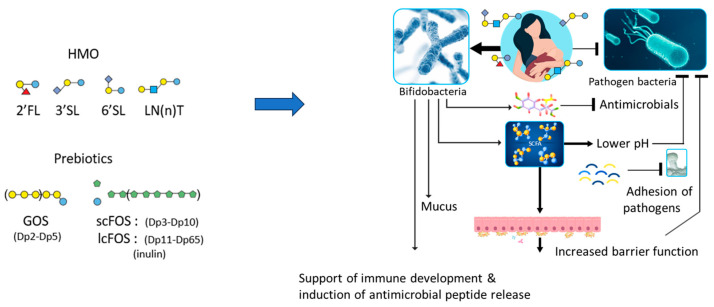
Effects of HMO and prebiotics often used in infant formulas on microbiota, SCFAs, epithelia, and the immune system.

As mentioned above, the effects of food components can also be indirect via steering the composition and metabolic activity of the intestinal microbiota. The role of the composition and metabolic activity of the gut microbiota in intestinal immune homeostasis and the role of the gut–lung axis are becoming clearer. A bifidobacteria-dominant intestinal microbiota that also contains relatively high levels of lactobacilli is associated with breastfeeding. Bifidobacterial and lactobacilli have enzymatic machinery to digest oligo- and polysaccharides that are not digested in the small intestine. Once these saccharides enter the colon, they are fermented by the microbiota, resulting in a growth advantage for Bifidobacteria and lactobacilli, and leading to the production of short chain fatty acids (SCFAs) as main metabolites. These SCFAs are anti-inflammatory components that support intestinal barrier function and also limit excessive immune activation [114,115]. In addition, SCFAs lower the pH in the colon, which limits the growth of certain bacterial species, such as *E. coli* (Figure 3).

The early development of the intestinal microbiota is important for immune maturation in infants. In the first weeks and months of life, the composition of the microbiota changes rapidly and becomes more established at around 2–3 yrs of life [116]. After one month of breastfeeding, a Bifidobacteria-dominant microbiota is established. The most important factor that modulates microbiota in early life are complex human milk oligosaccharides (HMOs) [106,117]. Up to 8% of the dry matter of breastmilk is milk oligosaccharides [117,118]. This is the third largest component in a mother’s milk.

There are more than 200 different milk oligosaccharides [106], with 2-fucosyl lactose (2′FL) being the most abundant oligosaccharide. In the early 2000’s, prebiotic oligosaccharides, especially galacto-oligosaccharides and fructo-oligosaccharides (GOSs and FOSs, respectively), were introduced in infant nutrition to mimic the Bifidobacteria-inducing effect of HMOs. Several studies have documented their effects on the prevalence of infections as well as on allergy development [15,119]. More recently, actual HMO-like 2′fucosylllactose and other HMOs have been added to infant nutrition as well [120,121].

Metabolites produced by the microbiota-like SCFA—rather than the bacteria themselves—are thought to mediate the main effects of HMOs on the infant’s immune system. Not only HMOs but also plant-derived fibers and non-digestible polysaccharides and prebiotic oligosaccharides all contribute to creating a diverse microbiota that supports the immune response and induces the production of metabolites, like SCFAs.

In addition to SCFAs, microbiota can also produce vitamins, bile acid metabolites, and aryl hydrocarbon receptor (AhR) ligands that support barrier function and immune function in the intestinal tract [50]. AhR ligands are mainly produced after the fermentation of tryptophan, which is present in high abundance in some milk proteins, like alpha lactalbumin, and can induce the production of antimicrobial peptides in the intestine. AhR ligands are also provided directly through ingestion of cruciferous vegetables (broccoli and cauliflower) [122] and even in breastmilk (quercitin) [123]. In addition to augmenting intestinal immune function, SCFAs are also important in preventing the development of (intestinal and also respiratory) allergies, as became clear from studies in which SCFA supplementation to mice could prevent the development of allergic airway inflammation [124]. In summary, establishing and maintaining a healthy intestinal microbiota is of importance for balanced immune development in infancy.

### 3.4. Other Immune Supportive Food Components

Polyunsaturated fatty acids are known to have immunomodulatory effects. The essential fatty acids α-linolenic acid and linoleic acid are needed in a diet to produce long-chain *n*-3 and *n*-6 polyunsaturated fatty acids (PUFAs). Typical *n*-3 fatty acids are eicosapentaenoic acid (EPA) and docosahexaenoic acid (DHA), which are present in fatty fish, and arachidonic acid (AA) is a typical *n*-6 PUFA that is present in plant oils. In addition to the already formed *n*-3 and *n*-6 PUFAs, a diet containing α-linolenic acid and linoleic acid is also important.

Whereas *n*-3 PUFAs have anti-inflammatory activity, the *n*-6 PUFA can have anti-inflammatory as well as pro-inflammatory activities. The right balance between *n*-3 and *n*-6 PUFA intake is important, as *n*-6 PUFA intake is often too high compared to *n*-3 intake. The recommended *n*-6/*n*-3 ratio is 4.6:1 [125,126]. Sufficient *n*-3 PUFA intake is therefore more limiting than sufficient *n*-6 intake.

In addition to the nutrients described above, other components can help protect against oxidative stress and have anti-inflammatory activity. These comprise a large group of mainly plant-derived phytochemicals, like flavonoids, polyphenols, carotenoids, as well as micronutrients [127]. Polyphenols are present in green tea, quercitin is a flavonoid, and β-carotene is an example of carotenoids that also function as a precursor to vitamin A.

Other mostly plant-derived immunomodulatory components that are not part of a normal diet but are available as food supplements (such as echinacea, ginseng, and β-glucans) are not included in this review but may also offer protection against infection.

### 3.5. Nutrition and Allergy

As described above, maternal nutrition can affect allergy development via transplacental nutrition delivery to the fetus, as well as by modifying breastmilk composition. In addition, nutrition in early life and in children’s diets can affect allergic outcomes [73]. For example, not only consumption of raw milk [97] but also some prebiotics [119] and probiotics [128] is associated with a reduced development of allergies, as is the intake of PUFAs [54,129] and diets high in fruit and fiber [130] and vitamins [131]. Recently, an allergy-preventive/managing immune-supportive diet approach has been proposed [132] that may play a role in allergy prevention if successfully implemented.

In addition to these normal dietary intakes, pre-and probiotics as well as micronutrients can easily be supplemented in a diet. Translation of the raw milk effect to prevent allergies will be dependent on the development of minimally processed infant nutrition products that are currently not available yet.

Further, the effect of processing of foods is becoming increasingly important, especially with the impact of glycation during processing [133,134,135]. Glycation is a process in which heat processing induces the formation of complex sugars linked to protein lysin. These changes not only make the protein less easily digestible (reducing efficiency of protein digestion and amino acid uptake) but also result in a stronger recognition of the potential food allergens by the immune system. A reduction in glycation by food processing or avoidance of highly glycated foods may contribute to reduced food allergy development in children as well as chronic inflammatory diseases (in adults and the elderly).

Finally, the timing of food intake has been shown to be crucial in the development of food allergies [136,137,138,139,140]. An early introduction of weaning foods at around 4 months has been shown to reduce the prevalence of food allergies, which has led to changes in dietary guidelines for infants supporting the introduction of foods from 4 months to 6 months of age. These and other important dietary guidelines for nutrition in infants and childhood are listed in Table A1.

## 4. Discussion

**Figure 4:**
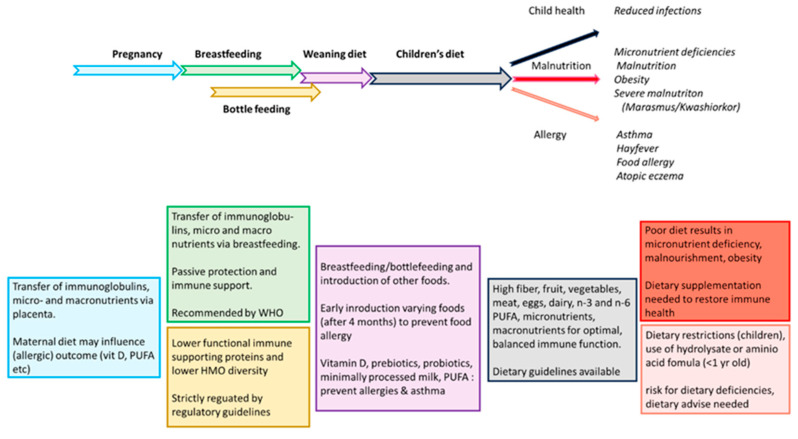
Schematic overview of nutrition and its effects in infancy and childhood.

This review describes our current knowledge on immune supportive effects of nutrition in early life to reduce the impact of infections and allergies in children (see Figure 4 for a summary).

In addition to what is described above, there are some important considerations that need to be made. For example, nutritional guidelines are based on recommended daily allowances (RDAs) for healthy people (adults). However, (chronic) infection increases the amount of nutrients needed, so RDAs may be too low for immune health in children with (chronic) infections and/or parasite infections. Further, even though breastfeeding is recommended for at least 6 months (WHO), it is very important that mothers should not have nutritional deficiencies or be malnourished and may need to supplement their diet with PUFAs and micronutrients to support the normal immune function of their children.

Not all mothers are able to breastfeed for various reasons. In such cases, infant formula can be given as a good alternative. This should contain all recommended nutrients and is tightly regulated to ensure an optimal composition (see Table A1). As a result, both breastfed and bottle-fed children should have a sufficiently high intake of dietary components to support healthy growth as well as immune protection. This is crucially important because breastfeeding and infant nutrition are the main sources of nutrition in early life.

The challenge comes when toddlers and young children need to receive their nutrients from a normal diet. In the first year of life, a child’s diet is dominated by breastfeeding and milk-based infant nutrition, but later in childhood, a full diet must be provided, with a balanced composition of enough micro- and macronutrients, as well as additional food components that have been described in nutritional recommendations for children (Table A1). The implementation of a healthy immune-supporting diet for children will be dependent on the availability of a varied diet, the economic capacity of the parents to provide this, as well as the knowledge level of the parents.

## 5. Conclusions

In conclusion, the current narrative review summarized current knowledge of the role of nutrition on the immune system and to prevent infections and allergies and non-communicable diseases. Our knowledge on dietary components that can support immune function, and the mechanisms via which they deliver these effects has increased tremendously over the last few decades, leading to a general consensus that a healthy immune supportive diet has to be more varied and contain high-quality proteins, PUFAs, micronutrients, and macronutrients. If the normal diet cannot sufficiently provide these nutrients, supplementation of these components (e.g., micronutrients and PUFAs) is recommended.

## Data Availability

No new data were created or analyzed in this study. Data sharing is not applicable to this article.

## Appendix A

**Table A1:** The table provides a link to nutritional guidelines for early-life nutrition, children’s nutrition, general dietary guidelines, and health claim regulations.

Institution	Type of Guideline	Hyperlink *
**INFANT AND YOUNG CHILD FORMULA GUIDELINES:**		
ESPGHAN	Infant Formula	https://onlinelibrary.wiley.com/doi/epdf/10.1097/01.mpg.0000187817.38836.42
ESPGHAN	Young child formula	https://onlinelibrary.wiley.com/doi/epdf/10.1097/01.mpg.0000187817.38836.42
EU	Legislation	https://eur-lex.europa.eu/eli/dir/2006/141/oj
USA	Infant Formula	https://www.fda.gov/food/infant-formula-guidance-documents-regulatory-information/regulations-and-information-manufacture-and-distribution-infant-formula#manufacture
CA	Infant Formula	https://www.canada.ca/en/health-canada/services/food-nutrition/legislation-guidelines/guidance-documents/infant-formula-human-milk-fortifier/guide-preparation-infant-formula-human-milk-fortifier-premarket-submissions.html
**OTHER NUTRITIONAL INFOR-MATION FOR CHILDREN**		
EU	Food for infants & young children	https://food.ec.europa.eu/safety/labelling-and-nutrition/specific-groups/food-infants-and-young-children_en#cereals-and-other-baby-foods
WHO/UNICEF	Global strategy for infant and young child feeding	https://iris.who.int/bitstream/handle/10665/42590/9241562218.pdf?sequence=1
CDC (USA)	Childhood Nutrition Facts	https://www.cdc.gov/school-nutrition/facts/?CDC_AAref_Val=https://www.cdc.gov/healthyschools/nutrition/facts.htm
DGA	Dietary guidelines for americans (US gov)	https://www.dietaryguidelines.gov/2025-advisory-committee-report
British Nutrition Foundation	Nutrition for Children	https://www.nutrition.org.uk/nutrition-for/children/
**GENERAL DIETARY GUIDELINES**		
FAO (UN)	Dietary guidelines(per region in the world)	https://www.fao.org/nutrition/education/food-dietary-guidelines/regions/countries/india/en/#:~:text=Promote%20exclusive%20breastfeeding%20for%206,plenty%20of%20vegetables%20and%20fruits
**HEALTH CLAIMS:**		
EU	Nutrition and health claims	https://food.ec.europa.eu/food-safety/labelling-and-nutrition/nutrition-and-health-claims_en
EFSA	Nutrition and health claims	https://www.efsa.europa.eu/en/topics/health-claims-art-13

* All hyperlinks were accessed on 27 March 2025.
